# MicroRNAs miR-19, miR-340, miR-374 and miR-542 regulate MID1 protein expression

**DOI:** 10.1371/journal.pone.0190437

**Published:** 2018-01-02

**Authors:** Kristoffer Unterbruner, Frank Matthes, Judith Schilling, Rohit Nalavade, Stephanie Weber, Jennifer Winter, Sybille Krauß

**Affiliations:** 1 Regulatory RNA-protein interactions in neurodegenerative diseases, German Center for Neurodegenerative Diseases (DZNE), Bonn, North Rhine-Westphalia, Germany; 2 Institute of Human Genetics, University Medical Center, Johannes Gutenberg University Mainz, Mainz, Rhineland-Palatinate, Germany; 3 Focus Program of Translational Neurosciences, Johannes Gutenberg University Mainz, Mainz, Rhineland-Palatinate, Germany; Universitat des Saarlandes, GERMANY

## Abstract

The MID1 ubiquitin ligase activates mTOR signaling and regulates mRNA translation. Misregulation of MID1 expression is associated with various diseases including midline malformation syndromes, cancer and neurodegenerative diseases. While this indicates that MID1 expression must be tightly regulated to prevent disease states specific mechanisms involved have not been identified. We examined miRNAs to determine mechanisms that regulate MID1 expression. MicroRNAs (miRNA) are small non-coding RNAs that recognize specific sequences in their target mRNAs. Upon binding, miRNAs typically downregulate expression of these targets. Here, we identified four miRNAs, miR-19, miR-340, miR-374 and miR-542 that bind to the 3’-UTR of the MID1 mRNA. These miRNAs not only regulate MID1 expression but also mTOR signaling and translation of disease associated mRNAs and could therefore serve as potential drugs for future therapy development.

## Introduction

The RING finger protein MID1 is involved in fundamental cellular processes including somatic cell growth and proliferation as well as neuron function (reviewed in [1]). Acting as E3 ubiquitin ligase MID1 marks the mTOR antagonist protein phosphatase 2A (PP2A) for degradation by the proteasome and thereby enhances mTOR activity [2]. Furthermore, MID1 assembles a ribonucleoprotein complex and regulates translation [3–6].

Germline mutations in *MID1* cause Opitz BBB/G syndrome (OS), a rare monogenic disorder involving malformations of the ventral midline including hypertelorism and hypospadias among others. Besides its role in OS MID1 function has been associated with the development and progression of various other diseases including cancer and neurodegenerative diseases. MID1 is overexpressed in certain cancer types and promotes cancer growth [7, 8]. In the brain, MID1 binds to and induces translation of pathologically expanded CAG repeat mRNAs, which are the cause for neurodegenerative diseases such as Huntington’s disease and spinocerebellar ataxias [5, 6]. Reducing the expression of MID1 is a promising new option to treat these diseases.

MiRNAs are endogenously expressed short (~20 nucleotide long) non-coding RNAs that base-pair their mRNA targets with imperfect complementarity (reviewed in [9]). The so-called “seed region” comprising nucleotides 2–8 of the miRNA, however, shows perfect complementarity and is important for target recognition. MiRNA binding sites are often located in the 3’-untranslated region (3'-UTR) of their target mRNAs [10–12]. Binding of a miRNA to its target mRNA can either cause degradation or inhibit translation.

Mimics of miRNAs that target MID1 could be promising miRNA therapeutics to treat cancer as well as neurodegenerative diseases. Whether MID1 is subject to miRNA targeting was, however, unknown. Here, we identified four miRNAs, miR-19, miR-340, miR-374 and miR-542 that bind the 3’-UTR of MID1 mRNA and inhibit MID1 protein production.

## Materials and methods

Human brain tissue was collected and stored as previously described [13]. Tissue was obtained with the families’ full consent and with the approval of the Leiden University Medical Center institutional Ethics Committee.

### Prediction of miRNA binding sites

MiRNA binding sites in the MID1 mRNA (Human MID1 ENST00000453318.2) were predicted using TargetScanHuman 6.2 (http://www.targetscan.org).

### Constructs

The first 1352 nucleotides of the MID1 3'-UTR containing the predicted binding sites of the above-mentioned miRNAs were cloned into the psiCHECK-2 vector (Promega) downstream of the renilla luciferase gene using the restriction enzymes NotI and XhoI.

### MiRNA mimics and inhibitors transfections and luciferase reporter assays

Chemically synthesized double-stranded RNAs mimicking mature endogenous miRNAs were transfected into HEK293T cells (ATCC) or HEK293T cells stably expressing HTT-exon1 with 51 CAG repeats [14] or into SH-SY5Y cells (ATCC). 10^5^ cells per well of a 24-well plate were seeded one day prior transfection. 2.5 μl per well of a 20 μM stock of miRNA mimics (hsa-miR-374a-5p, hsa-miR-542-3p, hsa-miR-216a-5p, hsa-miR-19b-3p, and hsa-miR-340-5p miScript miRNA Mimics, Qiagen) or miRNA inhibitors (hsa-miR-216a-5p, hsa-miR-340-3p, hsa-miR-374a, and hsa-miR-542-3p inhibitors from Qiagen, hsa-miR-19b-3p inhibitor from Sigma Aldrich (HSTUD0344)) were transfected using Oligofectamine (Invitrogen/Thermo Fisher Scientific) according to the manufacturer’s instructions. Twenty-four hours after transfection with mimics or inhibitors cells were transfected with plasmid DNA using Lipofectamine 2000 (Invitrogen/Thermo Fisher Scientific) according to the manufacturer’s instructions. Twenty-four hours later cells were lysed in 1x PLB (Promega) and protein concentration was measured by following the Qubit™ Protein Assay kit (Thermo Fisher Scientific). Samples were diluted to a concentration of 1 μg/μl. 10 μl of the diluted lysate was pipetted into a 96-well plate and luciferase assay was performed using the Dual-Luciferase® Reporter Assay System kit (Promega) following the manufacturer’s instructions. Measurement was performed in a FLUOstar Omega plate reader (BMG Labtech).

### Western blot

Cells were transfected with miRNA mimics, inhibitors, MID1 siRNAs (pool 5 siRNAs: TTGAGTGAGCGCTATGACAAA, AAGGTGATGAGGCTTCGCAAA, TAGAACGTGATGAGTCATCAT, CACCGCAUCCUAGUAUCACACTT, CAGGAUUACAACUUUUAGGAATT) or non-silencing control siRNAs (AATTCTCCGAACGTGTCACGT) and lysed as described above. After addition of 4x SDS PAGE buffer (EDTA 50 mM, Tris 200 mM, glycerol 40%, SDS 8%, β-mercaptoethanol 4%, bromophenol blue 0.008%) samples were boiled for 10 min at 95°C and proteins were analyzed on 10% SDS gels and blotted onto PVDF membranes (Roche). Blots were blocked in milk and incubated with the following antibodies: anti-actin (Abcam, rabbit), HRP-anti-rabbit (Cell signaling). For production of polyclonal MID1 antibodies MID1-peptides were synthesized (amino acids 84–113) and used for immunisation of rabbits (PINEDA). Eight weeks after immunisation high-titre sera were collected and affinity purified using the peptide coupled to SulfoLink Coupling Resin (Thermo Scientific) following the manufacturer’s instructions. The purified antibodies were validated on western blots of lysates from MID1 knockdown cells, as well as in western blot experiments in which peptide-blocking was performed (S1 Fig). The resulting bands were quantified using ImageJ software. Statistical analyses were performed using one-way ANOVA with post hoc Dunnett’s test to accommodate for multiple comparisons or Student’s *t*-test for two-group comparisons, as appropriate.

### Quantitative real-time PCR (qPCR)

Total RNA was isolated using the RNeasy Plus Mini Kit (Qiagen) according to the manufacturer’s instructions. cDNA was synthesized using the TaqMan reverse transcription reagents kit (Applied Biosystems) and qPCR was carried out using the SYBRGreen PCR master mix (Applied Biosystems). Primers used: GAPDH forward: CCACCCATGGCAAATTCC, GAPDH reverse: TGGGATTTCCATTGATGACAAG, MID1 forward: CTGCCAGGTCTGGTGTCATG, MID1 reverse: AATCAGGCTTAGGGCCCTTCT.

### Reverse transcription PCR (RT-PCR)

To detect miRNAs in HEK293T cells, a miRNA-enriched fraction was purified using the miRNeasy Mini and MinElute Cleanup Kits (Qiagen) according to the manufacturer’s instructions. MiRNAs were subjected to poly(T) adaptor reverse transcription as described (Shi et al., 2012) using *E*. *coli* poly(A) polymerase (New England Biolabs) and the TaqMan reverse transcription kit (Applied Biosystems) in a combined reaction. MiRNA sequences were amplified by PCR using miRNA-specific forward primers and the described universal poly(T) adaptor reverse primer. Primers used: hsa-miR-19b-3p-fwd: TGTGCAAATCCATGCAAAACTGA, hsa-miR-216a-5p-fwd: TAATCTCAGCTGGCAACTGTGA, hsa-miR-340-5p-fwd: GCTTATAAAGCAATGAGACTGATT, hsa-miR-374a-5p-fwd: CGTTATAATACAACCTGATAAGTG, hsa-miR-542-3p-fwd: GCTGTGACAGATTGATAACTGAAA.

## Results

### MiRNAs hsa-miR-374a-5p, hsa-miR-542-3p, hsa-miR-216a-5p, hsa-miR-19b-3p, and hsa-miR-340-5p target the MID1 3’-UTR at specific sites

**Fig 1:**
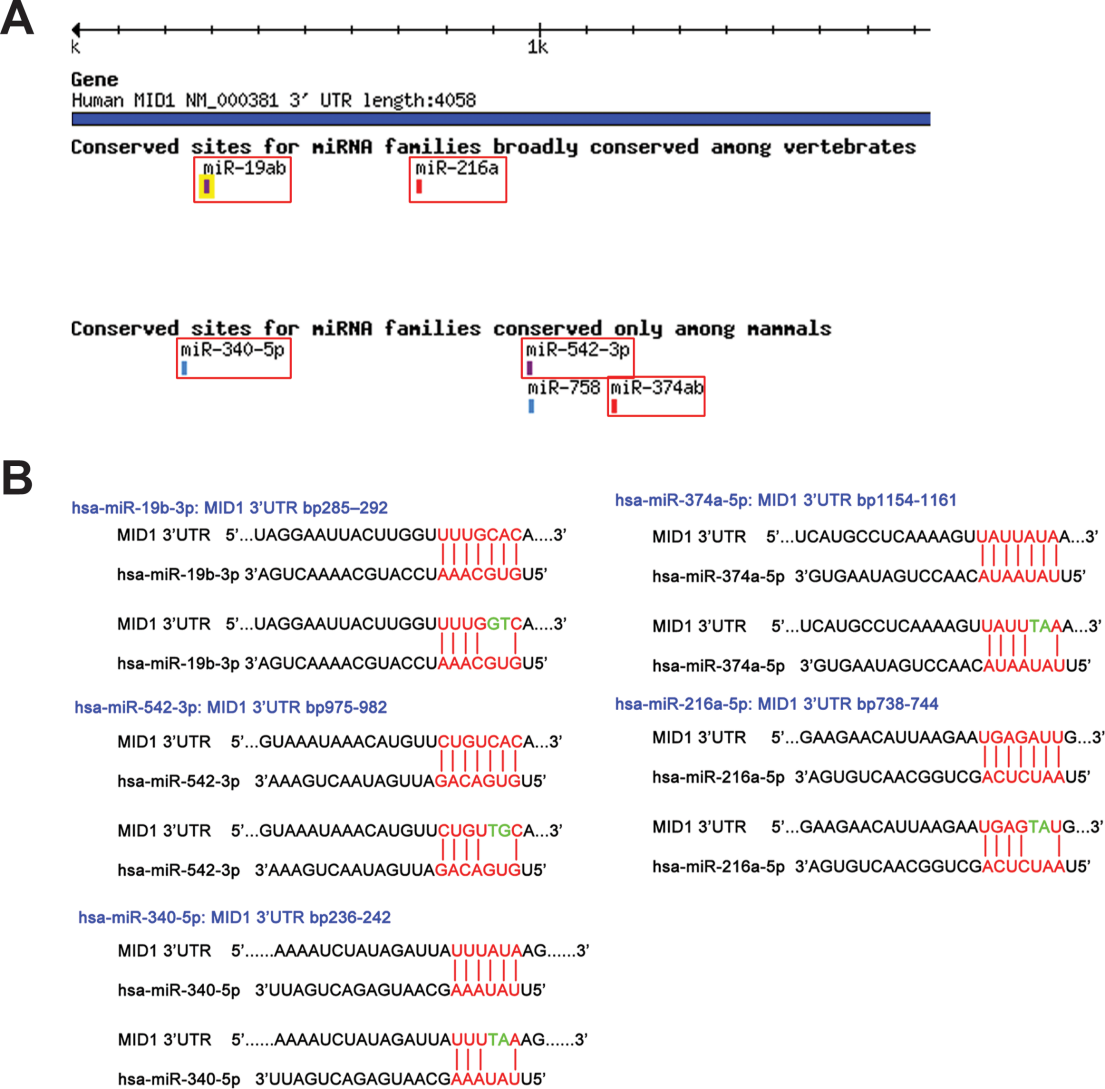
MiRNA binding sites in the 3’-UTR of MID1. (A) Targetscan prediction of miRNAs targeting within the first 1800bp of the MID1 3’-UTR. (B) Schematic drawing of the mutations that were inserted into the MID1 3’-UTR luciferase reporter constructs to mutate the seed region of the specific miRNAs.

The software Targetscan predicted several miRNAs to target the MID1 3'-UTR (Fig 1A). Because the MID1 3'-UTR is subject to several alternative polyadenylation events we only picked miRNAs that were predicted to target sequences in the 5'-part of the MID1 3'-UTR for further analysis. Furthermore, we restricted our analysis to miRNAs whose predicted binding sites in the MID1 3'-UTR were highly conserved in vertebrates (has-miR-19b-3p, has-miR216a-5p) or mammals (hsa-miR-374a-5p, hsa-miR-542-3p, hsa-miR-340-5p).

To test for binding of the miRNAs to the MID1 3'-UTR a luciferase reporter vector containing the first 1352 nucleotides of the MID1 3'-UTR cloned downstream of the renilla luciferase together with a pool of miRNA mimics or miRNA inhibitors was transfected into HEK293T cells and luciferase activity was measured. The pools were comprised of mimics or inhibitors of all five miRNAs. Firefly luciferase expressed from the same plasmid as renilla luciferase was used for normalization. Endogenous expression of these five miRNAs in HEK293T cells has been reported previously (miRmine Human miRNA Expression Database, [15]) and we validated this finding by reverse transcription PCR (S2 Fig).

**Fig 2:**
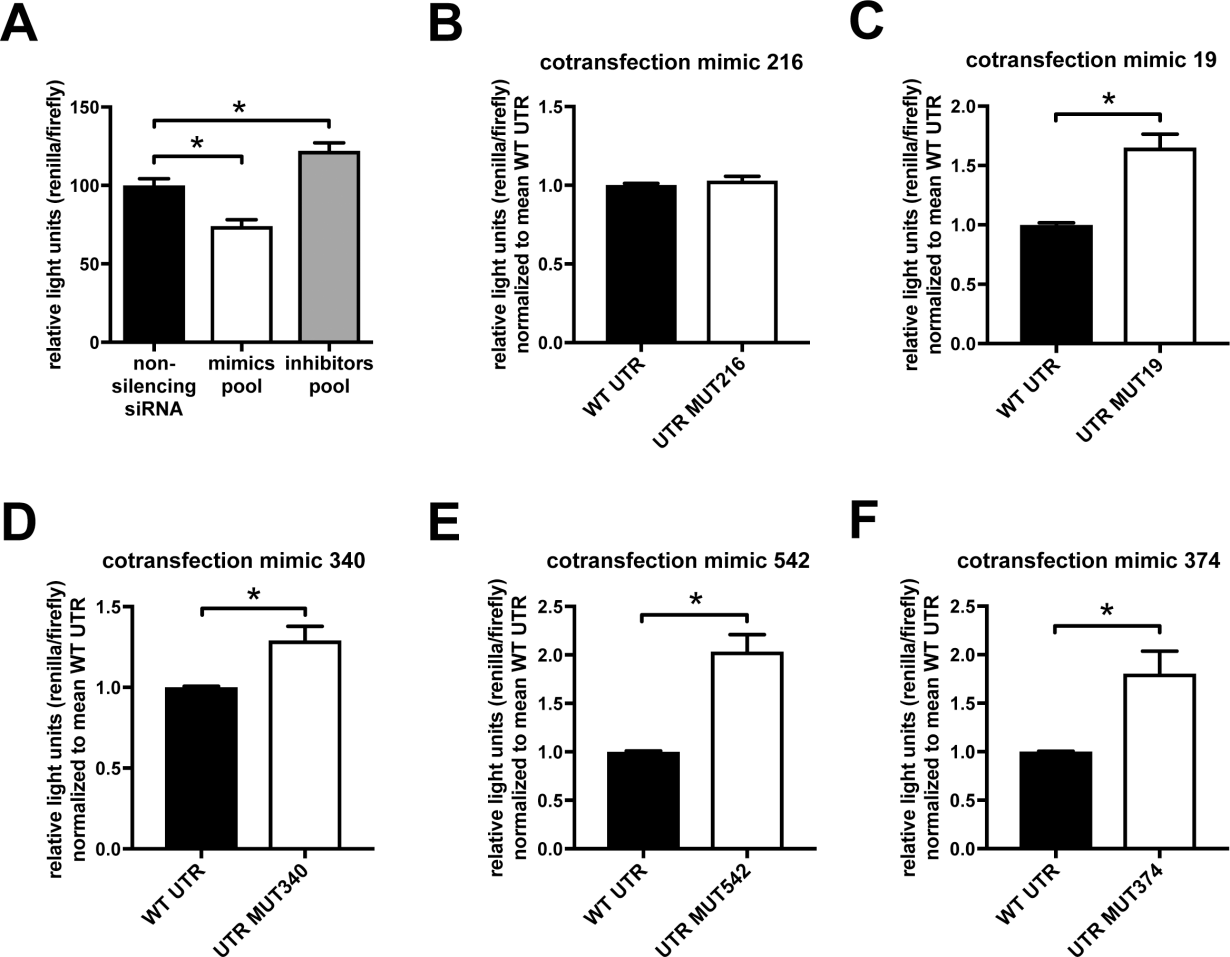
MiRNAs hsa-miR-374a-5p, hsa-miR-542-3p, hsa-miR-216a-5p, hsa-miR-19b-3p, and hsa-miR-340-5p target the MID1 3’-UTR. (A) HEK293T cells were transfected with a pool of miRNA mimics (hsa-miR-374a-5p, hsa-miR-542-3p, hsa-miR-216a-5p, hsa-miR-19b-3p, and hsa-miR-340-5p), a pool of miRNA inhibitors (hsa-miR-374a-5p, hsa-miR-542-3p, hsa-miR-216a-5p, hsa-miR-19b-3p, and hsa-miR-340-5p), or a non-specific control siRNA. 24 hours later the same cells were transfected with a reporter construct carrying renilla luciferase fused to the MID1 3’-UTR sequence (bp 1–1352) as well as firefly luciferase, which is used for normalization. Relative light units of renilla normalized to firefly luciferase are shown. Columns represent mean values +/- SEM (* p < 0.001, n = 13). (B-F) Cotransfection of HEK293T cells with miRNA mimics in combination with reporter constructs carrying renilla luciferase fused to the MID1 3’-UTR sequence (bp 1–1352), in which the seed regions of the specific miRNAs have been mutated (MUT). As in (A) firefly luciferase was used for normalization. The resulting data were normalized to the mean value for a control using the non-mutant (WT) MID1 reporter construct described in (A). Columns represent mean values +/- SEM (* p < 0.01, n = 18).

Transfecting the pool of miRNA mimics and inhibitors significantly decreased or increased, respectively, the activity of the MID1 luciferase reporter (Fig 2A), suggesting that miRNAs hsa-miR-374a-5p, hsa-miR-542-3p, hsa-miR-216a-5p, hsa-miR-19b-3p, and hsa-miR-340-5p indeed target the MID1 3’-UTR. To test each miRNA individually for its capacity to target the MID1 3’-UTR we mutated the respective miRNA binding sites in the MID1 luciferase reporter constructs (Fig 1B). These mutant constructs were co-transfected with the corresponding miRNA mimics and compared to transfections of the non-mutated constructs. A significant increase in luciferase activity could be observed upon transfection of all mutant constructs except for the construct containing the mutated miR-216 binding site (Fig 2B). These data show that miRNAs hsa-miR-374a-5p, hsa-miR-542-3p, hsa-miR-19b-3p, and hsa-miR-340-5p but not miR-216a-5p target the MID1 3’-UTR at the predicted sites.

### MiRNAs hsa-miR-374a-5p, hsa-miR-542-3p, hsa-miR-19b-3p, and hsa-miR-340-5p target endogenous MID1

**Fig 3:**
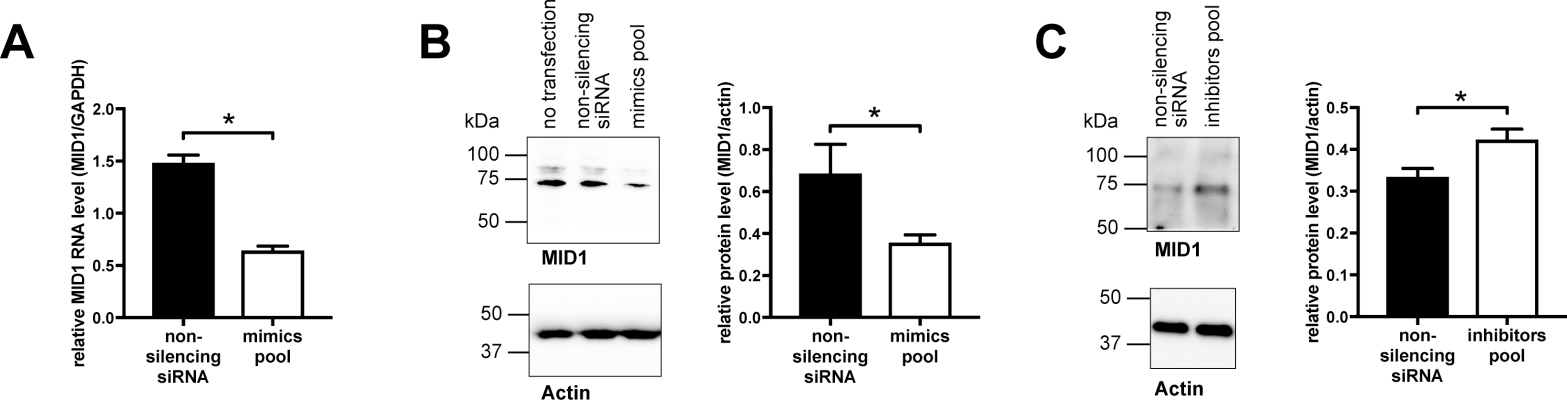
Micro RNAs hsa-miR-374a-5p, hsa-miR-542-3p, hsa-miR-19b-3p, and hsa-miR-340-5p target endogenous MID1. (A) HEK293T cells were transfected with a pool of miRNA mimics (hsa-miR-374a-5p, hsa-miR-542-3p, hsa-miR-19b-3p, and hsa-miR-340-5p), or a non-specific control siRNA. Relative MID1 mRNA expression was analyzed in a qPCR using MID1 specific primers and GAPDH-specific primers. Columns represent mean values +/- SEM (* p < 0.0001, n = 12). (B) HEK293T cells were transfected with a pool of miRNA mimics (hsa-miR-374a-5p, hsa-miR-542-3p, hsa-miR-19b-3p, and hsa-miR-340-5p), or a non-specific control siRNA. Left: MID1 protein levels were analyzed on a western blot using MID1 specific antibodies (upper blot) or Actin-specific antibodies (lower blot). A representative blot of n = 6 is shown. Right: quantification of blots. Columns represent mean values +/- SEM (* p < 0.05). (C) HEK293T cells were transfected with a pool of miRNA inhibitors (hsa-miR-374a-5p, hsa-miR-542-3p, hsa-miR-19b-3p, and hsa-miR-340-5p), or a non-specific control siRNA. Left: MID1 protein levels were analyzed on a western blot using MID1 specific antibodies (upper blot) or Actin-specific antibodies (lower blot). A representative blot of n = 6 is shown. Right: quantification of blots. Columns represent mean values +/- SEM (* p < 0.05).

To test if miRNAs hsa-miR-374a-5p, hsa-miR-542-3p, hsa-miR-19b-3p, and hsa-miR-340-5p target endogenous MID1, we transfected a pool of the corresponding miRNA mimics into HEK293T cells and analyzed the expression level of MID1 by qPCR and on western blots. In line with the above-mentioned luciferase reporter experiments, transfection of the miRNA mimics pool led to a significant reduction of MID1 expression at both RNA (Fig 3A) and protein level (Fig 3B). Consistently, transfection of miRNA inhibitors had the opposite effect and let to a significant increase in endogenous MID1 protein level (Fig 3C, S3 Fig, S4 Fig). Together these data indicate that miRNAs hsa-miR-374a-5p, hsa-miR-542-3p, hsa-miR-19b-3p, and hsa-miR-340-5p regulate MID1 expression.

### Targeting of MID1 by miRNAs hsa-miR-374a-5p, hsa-miR-542-3p, hsa-miR-19b-3p, and hsa-miR-340-5p reduces translation of MID1-target mRNAs

**Fig 4:**
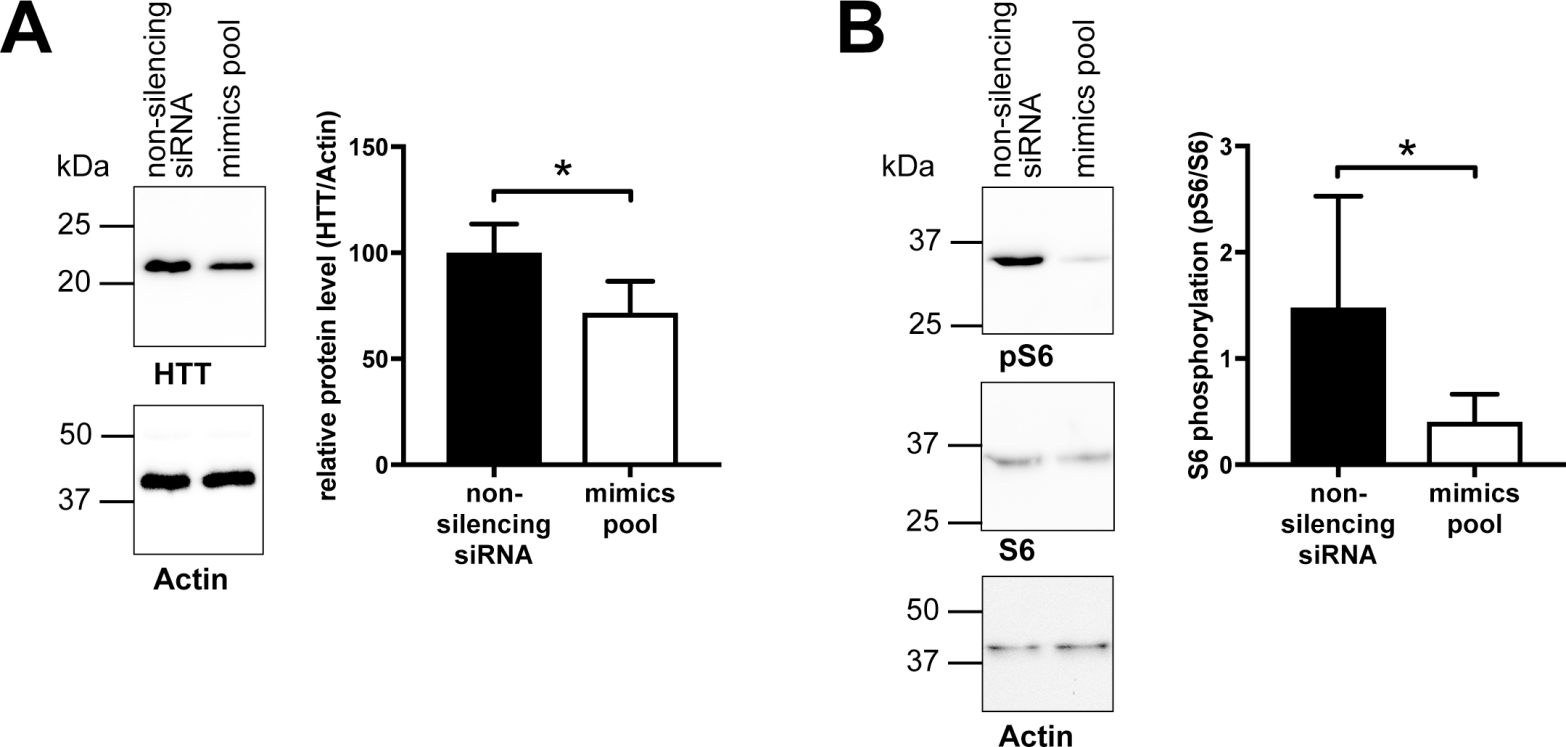
Targeting of endogenous MID1 by miRNAs hsa-miR-374a-5p, hsa-miR-542-3p, hsa-miR-19b-3p, and hsa-miR-340-5p leads to a reduction of HTT protein. HEK293T cells stably expressing HTT-exon 1 with 51 CAG-repeats were transfected with a pool of miRNA mimics (hsa-miR-374a-5p, hsa-miR-542-3p, hsa-miR-19b-3p, and hsa-miR-340-5p), or a non-specific control siRNA. Upper panel: Left: HTT-exon 1 protein levels were analyzed on a western blot using HTT specific antibodies or Actin-specific antibodies. A representative blot of n = 7 is shown. Right: quantification of blots. Columns represent mean values +/- SEM (* p < 0.05). Lower panel: phospho-S6 (pS6) as well as total S6 protein levels were analyzed on a western blot using specific antibodies. Actin was detected on the same blots as a loading control. A representative blot of n = 3 is shown. Right: quantification of blots. Columns represent mean values +/- SEM (* p < 0.05).

One previously described cellular function of the MID1 protein is the regulation of translation of its target mRNAs. MID1 induces translation of pathologically expanded CAG repeat mRNAs, such as huntingtin (HTT) mRNA [5, 6]. A miRNA-mediated reduction in MID1 should decrease protein levels of mutant HTT accordingly. To test this hypothesis, we transfected a previously described cell culture model for Huntington’s disease [14] with a pool of miRNA mimics (hsa-miR-374a-5p, hsa-miR-542-3p, hsa-miR-19b-3p, and hsa-miR-340-5p). These cells, a derivative of HEK293T stably expressing HTT-exon 1 with 51 CAG-repeats, were transfected either with the pool of MID1-targeting miRNA mimics or non-silencing control siRNA oligonucleotides. Cells were analyzed for HTT-exon 1 protein expression by western blot analysis. As expected, depletion of MID1 by miRNA mimics resulted in a significant reduction of HTT protein (Fig 4A, S5 Fig, S6 Fig).

MID1 inhibits PP2A, which in turn affects the phosphorylation of the translational regulator S6K and its target protein, the ribosomal subunit S6 [2, 5]. A decrease in MID1 expression leads to an upregulation of PP2A and dephosphorylation of S6K and S6. To test whether MID1-regulating miRNAs have an effect on S6 phosphorylation we measured S6 phosphorylation by western blot analysis upon transfection of the miRNA mimics pool containing mimics of hsa-miR-374a-5p, hsa-miR-542-3p, hsa-miR-19b-3p, and hsa-miR-340-5p. As expected expressing the miRNA mimic pool caused a strong reduction in S6 phosphorylation (Fig 4B, S7 Fig, S8 Fig).

**Fig 5:**
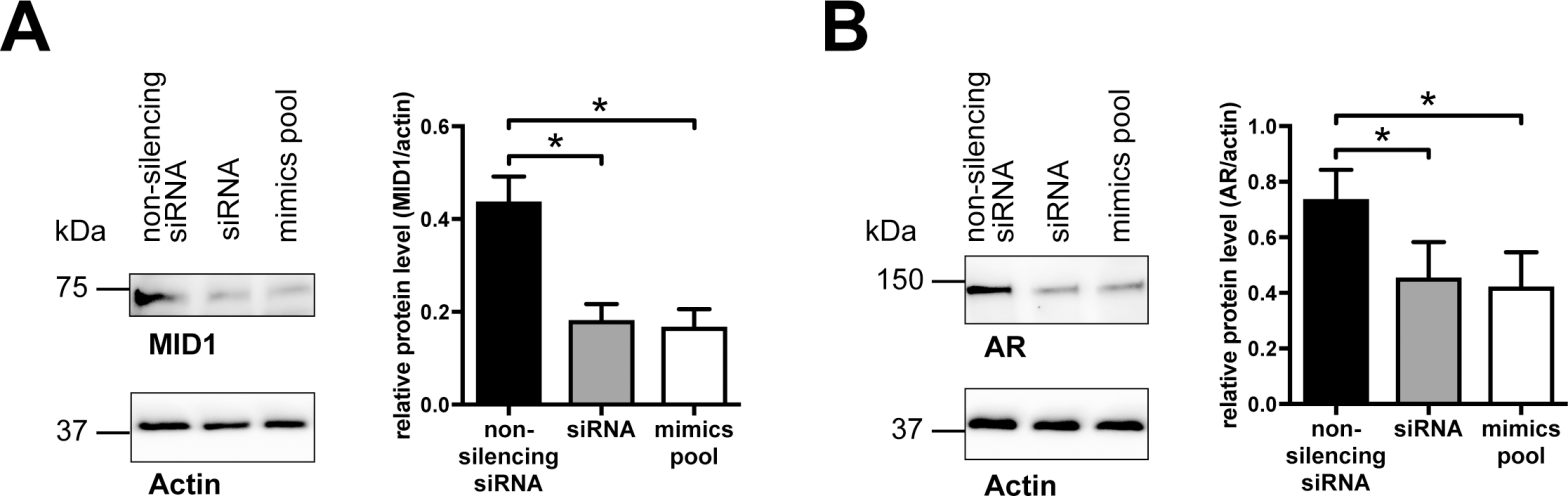
Targeting of endogenous MID1 by miRNAs hsa-miR-374a-5p, hsa-miR-542-3p, hsa-miR-19b-3p, and hsa-miR-340-5p leads to a reduction of AR protein. SH-SY5Y were transfected with a pool of miRNA mimics (hsa-miR-374a-5p, hsa-miR-542-3p, hsa-miR-19b-3p, and hsa-miR-340-5p), or MID1-specific siRNAs as positive control or a non-specific control siRNA as negative control. Upper panel: Left: MID1 as well as AR protein levels were analyzed on a western blot using specific antibodies. Actin was detected on the same membranes as loading control. A representative blot of n = 3 is shown. Right: quantification of blots. Columns represent mean values +/- SEM (* p < 0.05).

The androgen receptor (AR) mRNA is another important MID1 target [7]. To test if depletion of MID1 by miRNAs hsa-miR-374a-5p, hsa-miR-542-3p, hsa-miR-19b-3p, and hsa-miR-340-5p results in decreased AR protein levels, we transfected SH-SY5Y cells with the same pool of miRNA mimics as before. As positive control MID1-specific siRNAs and as a negative control non-silencing siRNAs were used. Cells were analyzed for MID1 and AR protein expression by western blot analysis. As expected, depletion of MID1 by miRNA mimics resulted in a significant reduction of AR protein (Fig 5, S9 Fig, S10 Fig, S11 Fig, S12 Fig). Taken together, these data show that depletion of MID1 by miRNAs hsa-miR-374a-5p, hsa-miR-542-3p, hsa-miR-19b-3p, and hsa-miR-340-5p leads to a reduction of several known MID1-targets: mutant HTT, S6 and AR.

## Discussion

**Fig 6:**
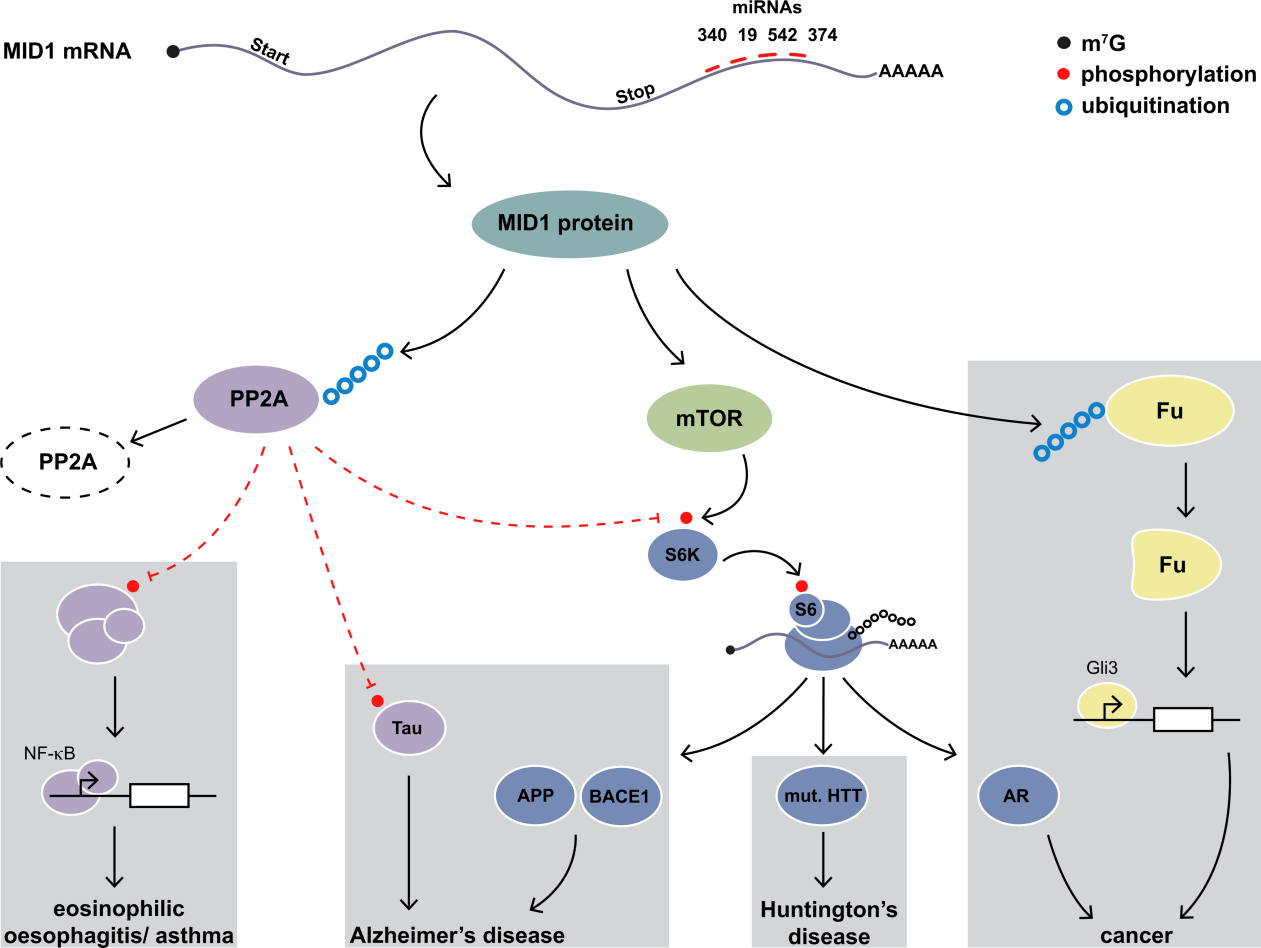
Model showing the role of MID1 in diverse diseases. We identified miRNAs hsa-miR-374a-5p, hsa-miR-542-3p, hsa-miR-19b-3p, and hsa-miR-340-5p as regulators of MID1. By controlling the expression levels of MID1 these miRNAs may affect several MID1-dependent processes. Functionally, MID1 acts as E3 ubiquitin ligase. Known targets of MID1’s ubiquitin ligase activity include PP2A and Fu. By catalyzing the ubiquitination PP2A MID1 induces the proteasomal degradation of PP2A, thereby reducing PP2A activity towards its target proteins. These include proteins involved in transcription regulation of inflammatory genes via NF-κB signaling, which are essentially involved in chronic inflammatory diseases such as asthma or eosinophilic oesophagitis, as well as the Tau protein, that is important in Alzheimer’s disease. Besides inhibiting PP2A MID1 stimulates the activity of mTOR. PP2A and mTOR regulate phosphorylation and thereby activity of the translational regulator S6K. Via PP2A and mTOR, MID1 controls translation of its target mRNAs. These include APP and BACE1, which play an important role in Alzheimer’s disease, mutant HTT, which causes Huntington’s disease, as we as that androgen receptor (AR), which is involved in prostate cancer. The second known target of MID1’s ubiquitin ligase activity is the kinase Fu. Upon MID1-dependent ubiquitination, this protein gets cleaved, which produces an active truncated protein that regulates the transcription factor GLI3.

In the embryo, MID1 is a dosage sensitive gene and is important for the development of ventral midline structures. In the adult, however, abnormal MID1 function has been associated with neurodegenerative diseases as well as cancer. Reducing MID1 expression in patients suffering from these diseases may be a promising therapeutic option. MiRNA-based therapeutics such as miRNA mimics could potentially be used to achieve such a downregulation of MID1 in disease state. Only one miRNA, miR-135b, has been shown previously to bind the MID1 3’-UTR and regulate MID1 expression in breast cancer cells [16]. Here we identified four additional miRNAs, hsa-miR-19b-3p, hsa-miR-340-5p, hsa-miR-374a-5p and hsa-miR-542-3p, that target the MID1 3'-UTR and regulate MID1 expression (Fig 6).

MID1 enhances the translation of mutant CAG repeat mRNAs and is therefore contributing to the neuropathology of polyglutamine diseases such as Huntington’s disease [5, 6]. Additionally, MID1 regulates processes that are linked to carcinogenesis and an abnormal activity of MID1 has been shown in different cancer cell types [7, 8]. Furthermore, MID1 expression is increased in certain types of cancer for example in prostate cancer cells [7]. Mechanistically, a carcinogenic action of MID1 can be explained by its interaction with proteins that play a role in cell cycle regulation. For example, MID1 regulates expression of *CyclinD1* via the transcription factor GLI3 [8, 17]. Furthermore, MID1 regulates activity of PP2A [2], a well established regulator of the cell cycle (reviewed in [18]). Whereas the four MID1-targeting miRNAs that we have identified have not been linked to Huntington’s disease so far, all of them have previously been linked to carcinogenesis [19–25]. Interestingly, expression of hsa-miR-19b-3p is down-regulated in gastric cancer [19] and hsa-miR-542-3p is decreased in several cancer types including oral squamous cell carcinoma [26], serous ovarian tumor [22], esophageal cancer [24] and colorectal cancer [25] and acts as a tumor suppressor miRNA. Future studies should investigate whether a downregulation of these miRNAs is functionally connected to an upregulation of MID1 in these cancers.

MicroRNAs have oftentimes more than one target and can regulate the expression of several genes. In addition to MID1, miRNAs hsa-miR-19b-3p, hsa-miR-340-5p, hsa-miR-374a-5p and hsa-miR-542-3p have other target mRNAs (Table 1). Several of these known target mRNAs are associated with carcinogenesis. For example, hsa-miR-19b-3p regulates PTEN, a tumor suppressor that is mutated in a large number of cancers [27], and ESR1, mutations in which are associated with breast cancer [28].

Hsa-miR-340-5p regulates expression of oncogenic target mRNAs. For example, MET is a proto-oncogene [45], KRAS is a proto-oncogene [46], and overexpression of RHOA leads to tumor formation [47]. Hsa-miR-542-3p acts as tumor suppressor by targeting survivin (BIRC5) [29, 30], the oncogene astrocyte-elevated gene-1 (MTDH) [38], or angiopoietin-2 (ANGPT2) that plays a role in tumor angiogenesis [41]. Hsa-miR-374a-5p interferes with carcinogenesis by regulating Srcin1, which contributes to the growth and metastasis of colorectal cancer [31], or by regulating WIF1 that acts as tumor suppressor [32]. Interestingly, we identified MID1 as the first common target of these four miRNAs.

**Table 1:** Targets of miRNAs hsa-miR-19b-3p, hsa-miR-340-5p, hsa-miR-374a-5p and hsa-miR-542-3p.

Experimentally validated miRNA target mRNAs				
miRNA:	hsa-miR-19b-3p	hsa-miR-340-5p	hsa-miR-374a-5p	hsa-miR-542-3p
target mRNA	ATXN1 [33]	ABCB5 [34]	CEBPB [35]	BIRC5 [36]
	BCL2L11 [37]	HNRNPA2B1 [38]	**MID1**	ILK [39]
	CUL5 [40]	KRAS [41]	PTEN [42]	MTDH [43]
	CYP19A1 [44]	LPAATβ [45]	SRCIN1 [46]	BMP7 [47]
	ESR1 [48]	MECP2 [41]	WIF1 [42]	RPS23 [23]
	GCM1 [44]	MET [49]	WNT5A [50]	ANGPT2 [51]
	**MID1**	**MID1**		FZD7 [52]
	MXD1 [53]	PTBP1 [38]		PIM1 [54]
	MYCN [55]	RHOA [56]		cortactin [57]
	PPP2R5E [37]	ROCK1 [58]		AKT1 [59]
	PRKAA1 [37]	SOX2 [60]		integrin-linked kinase [59]
	PTEN [61]	STAT3 [62]		PIK3R1 [59]
	ring finger protein 11 [63]			sphingosine-1-phosphate receptor 1 [64]
	SMAD4 [65]			UBE3C [66]
	SOCS1 [67]			**MID1**
	TGF-β R II [68]			
	TLR2 [69]			
	TNFAIP3 [70]			
	TP53 [71]			

Another interesting observation is that a significantly decreased expression of miR-19b-3p has been reported to coincide with increased protein expression of BACE1 in Alzheimer’s disease, although miR-19b-3p does not directly target BACE1 [72]. We show here that miR-19b-3p targets MID1, a protein that we have previously shown to induce protein expression of two mRNAs that are crucial for the development of amyloid plaques in Alzheimer’s disease, namely BACE1 [4] and APP [73]. Furthermore, we have observed increased expression of MID1 in Alzheimer’s disease brains [74]. Our data showing that miR-19b-3p targets MID1 provide one possible explanation for increased expression of MID1 in Alzheimer’s disease tissue, that may be caused by reduced levels of miR-19b-3p [72, 75]. Future studies should address if miRNA based therapeutics such as miRNA mimics of the four MID1-targeting miRNAs hsa-miR-19b-3p, hsa-miR-340-5p, hsa-miR-374a-5p and hsa-miR-542-3p could be used to downregulate MID1 in Alzheimer’s disease models and thereby counteract formation of amyloid plaques.

## Supporting information

S1 Fig Validation of the polyclonal MID1 antibody. (a) HEK293T cells were transfected with non-targeting (“control”) or MID1-specific siRNAs, lysed, and subjected to western blotting using the MID1 antibody for detection, either in absence (left) or presence (right) of the immunizing peptide (1 μg/ml). The antibody detects a specific band at ~75 kDa, that is reduced in MID1 knockout samples. Blocking with the immunizing peptide results in total loss of the specific signal. (b) Human temporal cortex lysate was subjected to western blotting using the MID1 antibody in absence or presence of the immunizing peptide. The antibody detects two specific bands.
(TIF)

S2 Fig MicroRNA expression in HEK293T cells. From HEK293T total RNA a miRNA-enriched fraction was prepared. RNAs were extended by a poly(A) tailing reaction followed by reverse transcription using a poly(T) adaptor primer. MiRNA sequences were amplified by PCR using miRNA-specific forward primers and a universal poly(T) adaptor reverse primer. Samples in which reverse transcriptase was omitted (No RT) were used as controls.
(TIF)

S3 Fig Full blot of the western blot shown in Fig 3C (detection anti-MID1 antibody). (TIF)

S4 Fig Full blot of the western blot shown in Fig 3C (detection anti-actin antibody). (TIF)

S5 Fig Full blot of the western blot shown in Fig 4A (detection anti-HTT antibody). (TIF)

S6 Fig Full blot of the western blot shown in Fig 4A (detection anti-actin antibody). (TIF)

S7 Fig Full blot of the western blot shown in Fig 4B (detection anti-pS6 antibody). (TIF)

S8 Fig Full blot of the western blot shown in Fig 4B (detection anti-S6 antibody). (TIF)

S9 Fig Full blot of the western blot shown in Fig 5A (detection anti-MID1 antibody). (TIF)

S10 Fig Full blot of the western blot shown in Fig 5A (detection anti-actin antibody). (TIF)

S11 Fig Full blot of the western blot shown in Fig 5B (detection anti-AR antibody). (TIF)

S12 Fig Full blot of the western blot shown in Fig 5B (detection anti-actin antibody). (TIF)

S1 Appendix Primary data of graphs shown in Figs 2, 3, 4 and 5. (XLSX)
