# Supplementary figures and images for: MicroRNAs miR-19, miR-340, miR-374 and miR-542 regulate MID1 protein expression
Source: PLoS One. 2018 Jan 2;13(1):e0190437. doi: 10.1371/journal.pone.0190437 (PMC5749791; doi:10.1371/journal.pone.0190437)

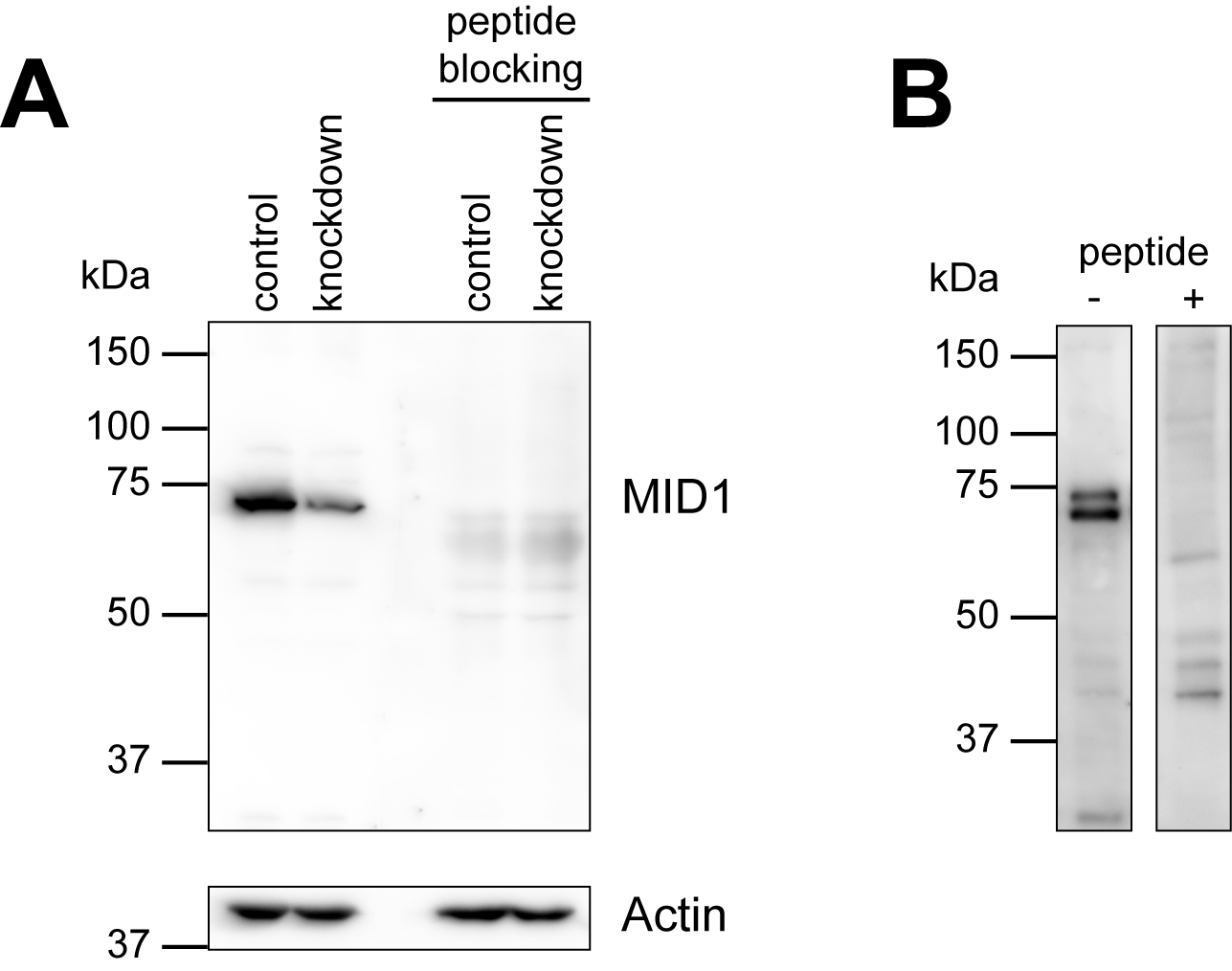

Supplement: S1 Fig — (a) HEK293T cells were transfected with non-targeting (“control”) or MID1-specific siRNAs, lysed, and subjected to western blotting using the MID1 antibody for detection, either in absence (left) or presence (right) of the immunizing peptide (1 μg/ml). The antibody detects a specific band at ~75 kDa, that is reduced in MID1 knockout samples. Blocking with the immunizing peptide results in total loss of the specific signal. (b) Human temporal cortex lysate was subjected to western blotting using the MID1 antibody in absence or presence of the immunizing peptide. The antibody detects two specific bands. (TIF) [file pone.0190437.s001.tif]

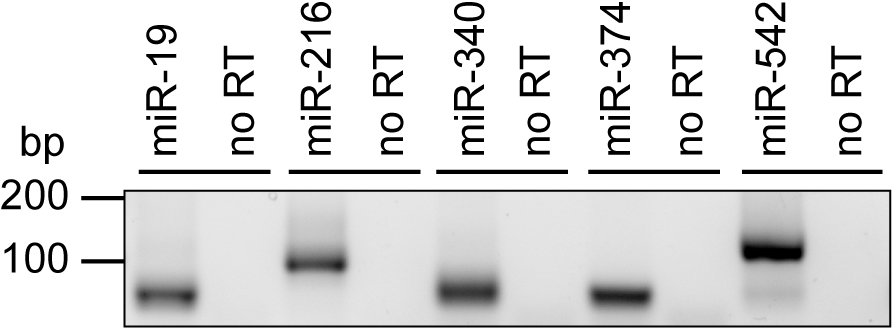

Supplement: S2 Fig — From HEK293T total RNA a miRNA-enriched fraction was prepared. RNAs were extended by a poly(A) tailing reaction followed by reverse transcription using a poly(T) adaptor primer. MiRNA sequences were amplified by PCR using miRNA-specific forward primers and a universal poly(T) adaptor reverse primer. Samples in which reverse transcriptase was omitted (No RT) were used as controls. (TIF) [file pone.0190437.s002.tif]

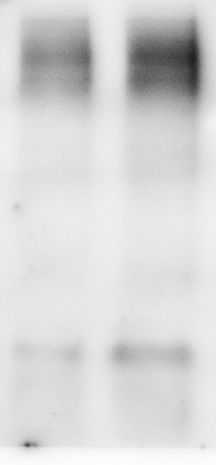

Supplement: S3 Fig — (TIF) [file pone.0190437.s003.tif]

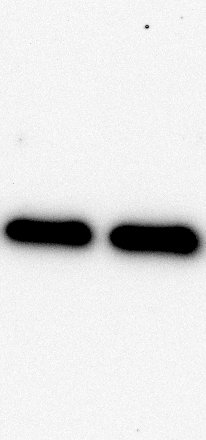

Supplement: S4 Fig — (TIF) [file pone.0190437.s004.tif]

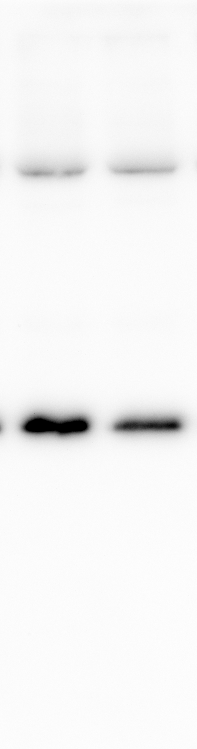

Supplement: S5 Fig — (TIF) [file pone.0190437.s005.tif]

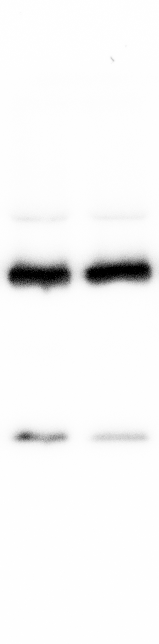

Supplement: S6 Fig — (TIF) [file pone.0190437.s006.tif]

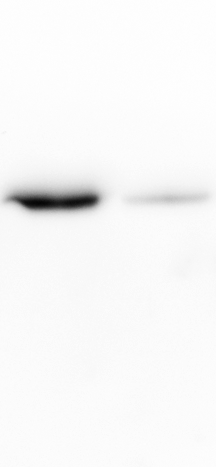

Supplement: S7 Fig — (TIF) [file pone.0190437.s007.tif]

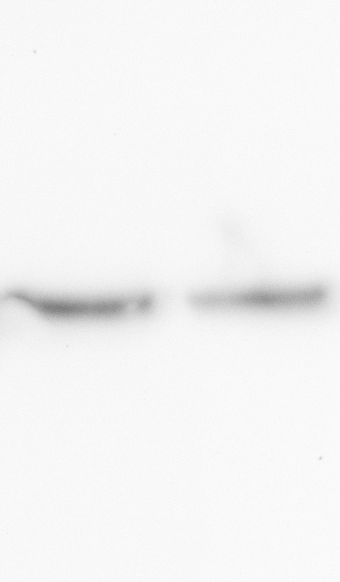

Supplement: S8 Fig — (TIF) [file pone.0190437.s008.tif]

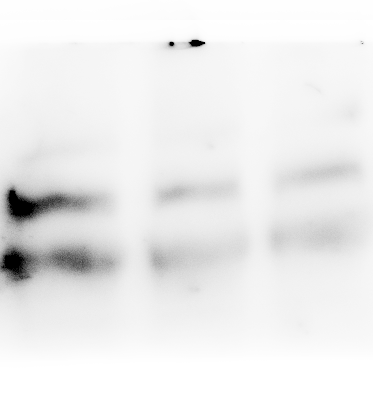

Supplement: S9 Fig — (TIF) [file pone.0190437.s009.tif]

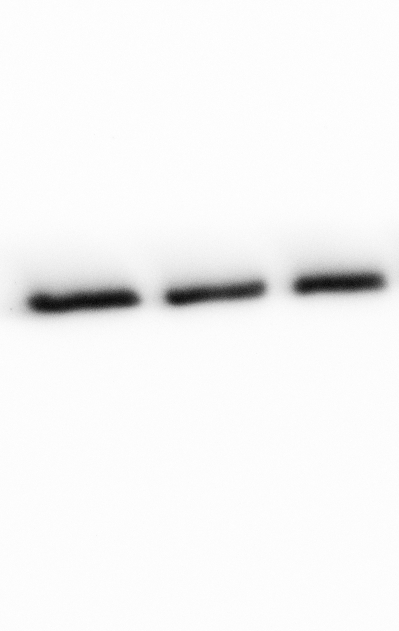

Supplement: S10 Fig — (TIF) [file pone.0190437.s010.tif]

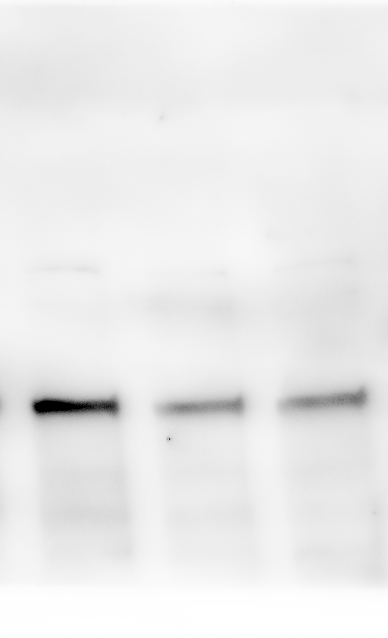

Supplement: S11 Fig — (TIF) [file pone.0190437.s011.tif]

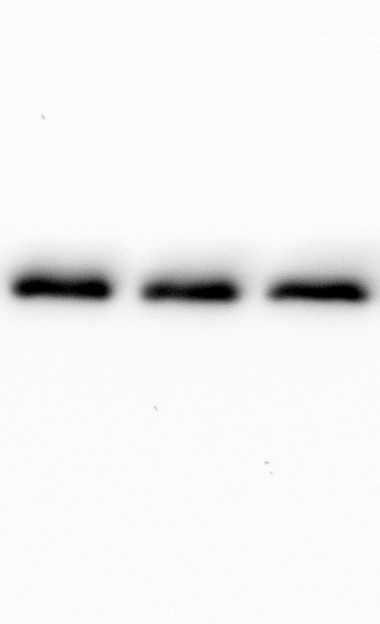

Supplement: S12 Fig — (TIF) [file pone.0190437.s012.tif]
